# Situating HIV Stigma in Health Facility Settings: A Qualitative Study of Experiences and Perceptions of Stigma in ‘Clinics’ among Healthcare Workers and Service Users in Zambia

**DOI:** 10.1177/23259582221100453

**Published:** 2022-05-16

**Authors:** Sanny Mulubale, Sue Clay, Corinne Squire, Virginia Bond, Kasoka Kasoka, Lucy Stackpool-Moore, Tessa Oraro-Lawrence, Mutale Chonta, Chipo Chiiya

**Affiliations:** 1 108234 The University of Zambia, MIET Africa, Lusaka, Zambia; 2 3C Regional Consultancy, Lusaka, Zambia; 3 School of Policy Studies, Bristol University, Bristol, England; 4 London School of Hygiene and Tropical Medicine, London, UK; 5 503988 Zambart, School of Public Health, University of Zambia, Lusaka, Zambia; 6 International AIDS Society, Geneva, Switzerland; 7 Watipa, Sydney, Australia

**Keywords:** ART, clinic, healthcare workers, HIV, stigma, Zambia

## Abstract

The study focused on the representations, processes and effects of HIV stigma for healthcare workers living with HIV within health facilities in Zambia. A descriptive study design was deployed. A total of 56 health workers and four service user participants responded to a structured questionnaire (n = 50) or took part in key informant interviews (n = 10) in five high HIV-prevalence provinces. Most participants did not disclose if they were living with HIV, except for four participants who responded to the questionnaire and were selected for being open about living with HIV. Semi-structured interviews were carried out with health workers in key government health facility positions. The questions were standardized and used a Likert scale. Descriptive statistical and thematic analyses were applied to the data. Results show that antiretroviral treatment (ART) has an impact on stigma reduction. Almost half the participants agreed that treatment is reducing levels of HIV stigma. However, fears of exposure of HIV status and labelling and judgemental attitudes persist. No comprehensive stigma reduction policies and guidelines in healthcare facilities were mentioned. Informal flexible systems to deliver HIV services were in place for health workers living with HIV, illustrating how stigma can be quietly navigated. Lack of confidentiality in healthcare facilities plays a role in fuelling disclosure issues and hampering access to testing and treatment. Stigma reduction training needs standardization. Further, codes of conduct for ‘stigma-free healthcare settings’ should be developed.

## Introduction

Stigma affects health outcomes, ranging from limiting health-seeking behaviours to lack of adherence or engagement in treatment and testing programmes by those affected.^1^ Stigma can also create social isolation and low performance, especially through workplace discrimination, and has powerful negative impacts on mental health that converge with issues of gender and social status.^2^ HIV-related stigma, therefore, makes it more difficult to stop new HIV acquisitions, sustain treatment adherence, promote mental health and reduce HIV-related deaths. People can become afraid to be tested, inform others that they are living with HIV and openly seek HIV care, including antiretroviral treatment (ART).^3^

Stigma can be defined as a social process that is characterized by stereotyping, labelling, gossip and isolation that can result in loss of social status and low self-esteem and lead to discrimination and poor health-seeking behaviour.^4^ In this study, we defined HIV-related stigma as manifestations of negative feelings, beliefs, attitudes and expressions towards people living with HIV and the internalization of these manifestations by people living with HIV to make them feel less about themselves; these manifestations are heightened by wider contexts of power and inequities.^5^ There are different types of HIV-related stigma, including perceived, structural, anticipated, enacted and internalized stigmaa, each of which is experienced and can be addressed differently.^6^

Stigma devalues an individual through labels that emanate from perceived ‘otherness’ and can cause social isolation and low self-esteem.^7^ A common example is in healthcare settings, where being seen at the clinic, for example, is often a trigger of internalized and anticipated stigma.^1^ This happens in spite of effective treatment breakthroughs that have rendered living with HIV as not a ‘death sentence’ but a chronic manageable illness.

Studies have shown that anticipated and structural stigma in health facilities hinders prevention efforts and early treatment.^8–10^ Stigma in health facilities can be manifested by denying or reducing the access of people living with HIV to healthcare services.^6^ Further, the fear of being stigmatized can, in turn, provoke people living with HIV to avoid or resolve to reduce contact with clinical services.^11^ Unwanted disclosures can be impediments to accessing health services created by stigma, which reduces the effectiveness of the HIV response; these disclosures can take place through being seen carrying ART or client cards, using demarcated HIV services (‘the ART block’), distinctive client flow and any associated items.^5,12^

Stigma can have psychosocial effects, as well as direct effects on behaviours. Stigma reduction, however, does not mean only the removal of adverse reactions from others towards a condition that requires ART. HIV stigma persists even when HIV is invisible in the form of social markers.^8,13,14^ Although ART is simply a pharmacological agent that places HIV between a treatable epidemic and a disappearing tragedy, the legacy of HIV history and the ongoing effects (social, cultural, economic and political) continue.^8,15^ Some stigma interventions are therefore part of the psychosocial goals of the struggle against HIV and are not aimed only at changing specific health behaviours directly. In the ART era, stigma has moved from experiences linked to symptomatic AIDS to other less direct signals that indicate that someone is living with HIV. Stigma directly affects what people do, for example, go for treatment or discriminate when providing treatment, and also how they and others feel about themselves, which, in turn, does affect what they do.

We wish to argue that the lack of knowledge of untransmissibility of HIV when successfully treated – of what has been labelled ‘Undetectable = Untransmittable (U = U)’ – contributes to internalized stigma in healthcare settings.^8^ This is in addition to myths and rumours that spread blame, fear and gossip about what it means to live with HIV. There seem to be generally held opinions, among others, that people living with HIV are morally blameworthy and also die earlier than those who are not living with HIV, even with successful treatment management*.* In this vein, stigma is dangerous because it generates prejudice, discrimination, isolation, anxiety and depression that is not based on factual information.^8,16^

Nonetheless, there is need to point out that stigmatizing attitudes and practices are not just restricted to the general public; people within public health settings and even people living with HIV also stigmatize. Identity protection plays an important part in understanding health workers living with HIV and their desire to not be identified as belonging to a group that is ideally ‘other’ than them.^17^ The separation of ‘us’ and ‘them’ is well established as a stigma process, dating back to Goffman and other social psychologists.^5^

The labelling of differences with negative attributes is founded on change processes that feed into the broader parallel separation processes of ‘us’ from ‘them’,^18^ and that characterizes HIV stigma processes in both healthcare and non-healthcare settings. Thus, even many health workers feel the need to hide their HIV status or they avoid accessing services because they do not want to be identified as ‘them’.^1,2^

## Zambia and HIV-Related Stigma in Healthcare: A Situational and Conceptual Account

This study explores healthcare workers’ and service users’ experiences and perceptions of stigma in clinical settings in Zambia. It was funded and commissioned with the purpose of reviewing current commitments towards reducing HIV-related stigma in Zambia. The country is making progress towards meeting the 2025 95-95-95 targets through, among other things, development of policy and legal provisions that protect people living with and affected by HIV.^19^ However, stigma and discrimination, including at healthcare facilities, continue to derail much-needed progress in Zambia. Among other factors, people still sometimes avoid testing for HIV and accessing treatment services due to inequalities that are embedded in the continuum of care in health facilities in high HIV-prevalence areas.^4,20^

Health workers and facility settings play an important role in mitigating and/or exacerbating HIV stigma. Although stigma is reported to be low in hospitals,^21^ people living with HIV in Zambia have reported exclusion from healthcare facilities.^22,23^ Moreover, even though anti-stigma policies are available, such policies are not implemented in practice.^24^ Currently, the burden of HIV stigma is recognized as a phenomenon that changes and sometimes overlaps with other concepts, such as acceptance or identity in representations of living with HIV.^7^ A validated set of items for people living with HIV captured internalized stigma and experience measures that demonstrated that 22.0% of people living with HIV experienced stigma in the community and 7.1% in healthcare settings.^9^

There are hardly any studies carried out in Zambia on the impact of laws and policies on universal access to healthcare and barriers to the HIV continuum of care for key populations, such as men who have sex with men, prisoners, sex workers, trans people and people who inject drugs. Similarly, there seem to be no studies related to healthcare workers’ values, attitudes and behaviours associated with the aforementioned key population. Best-practice aspects of stigma-related research have been recommended in the literature, but rarely carried out. Most indicators in stigma-related work measure socio-cognitive aspects of HIV stigma and do not address the implications of stigma in healthcare facilities on HIV treatment and prevention in Zambia. And these indicators are mainly developed overseas.^8^ This study sought to address some of these gaps in the literature about healthcare settings in Zambia, particularly the impact of ART on stigma reduction and local strategies to address stigma in healthcare.

This study offered an opportunity to carry out a situation analysis of HIV status stigma in health facility settings among healthcare workers and a small number of service users living with HIV. It reports and examines findings on the experiences of HIV status and perceptions of healthcare workers and service users with HIV on stigma in health facilities. Given that most studies have focused on community-level stigma, this study is concerned with healthcare settings because stigma at point of care can hinder HIV responses that are effective.

## Methods

### Study Design, Population and Demographics

This was a mixed methods^25^ study that used a descriptive design in evaluating HIV stigma manifestations in healthcare facilities among healthcare workers, some of whom might also be service users with HIV. It is not easy to identify healthcare workers living with HIV and many of them do not disclose their status openly, including to researchers. Therefore, because we could not openly include health workers living with HIV who shared their status, we also included four service users who were not healthcare workers and were open about living with HIV. Interviews with key informants were done to generate more comprehensive accounts of the issues. These interviews also allowed follow-up questions and further probing for detailed insights. The questionnaires for both service users and healthcare workers provided lenses through which perceptions and experiences of stigma at health facilities were explored. The inclusion or focus on service users, besides healthcare workers, enabled a wider range of information sources from different points of view because HIV stigma perceptions matter and affect both healthcare workers and service users.

### Sample

A total of 60 participants (42 women, 14 men and four trans people) were recruited using purposive and convenience sampling. Participants were between the ages of 18 and 55 years. From the 60, we selected 10 individuals in positions of authority, such as sisters-in-charge of a clinic or HIV unit in health facilities, four service users and 46 healthcare workers, which included nurses and other general administrative officers working in healthcare setting. People in this sample were selected on the basis that they worked in HIV care services, except for the four service users. The healthcare workers were not recruited as people living with HIV. But some of them may have been HIV positive or were directly affected; hence, claims about the patterns of behaviour and experiences for and by healthcare workers living with HIV were drawn and discussed, as will be noticed in sections below. Among the participants and perhaps just for this study, three participants in questionnaires selected bisexual as their sexual orientation, a disclosure that is uncommon in Zambia.

### Study Setting

The sites of the study included five provinces (Lusaka, Central, Copperbelt, Western and Southern). Researchers visited one facility in each province for the study. Three health facilities were in peri-urban areas and two were in urban high-density settings. The majority of participants in all the health facilities in this study came from lower-income populations. Participants were asked to join the study through the health facility management. Table 1 shows the numbers and demographic characteristics of participants across data collection sites.

### Data Collection and Analysis

Mixed methods conventionally means qualitative and quantitative, a recommended approach for stigma research (see Mulubale, Rohleder and Squire).^2^ This approach was utilized by seeking written structured responses through the questionnaires*,* which were susceptible to a descriptive statistical analysis, and verbal associations and semi-structured responses within the interviews. Key informant interviews with the 10 people in positions of power within the facility were helpful because some questions required feedback from people in management roles. The other 50 participants then completed questionnaires.

The questionnaire questions were standardized and generated out of interviews and the study's specific research questions, as well as from the stigma index framework developed by Mahendra and colleagues.^3^ The type of responses required Likert scale answers, and there was an open question at the end that led to qualitative data, to which thematic analysis was also applied.

Participants were briefed on the research and given documents, including an information sheet, written informed consent form and demographics information sheet. All documents were explained to the interviewees before the interviews and completion of questionnaires. Some questionnaire instructions required explanation. The completion of each questionnaire for the 50 participants took 25 to 40 minutes. The duration of semi-structured interviews ranged from one to two hours for the initial 10 key informants. The interviews took place in offices of each of these key informants and the settings provided privacy.

The audios for semi-structured interviews with the 10 key informants were transcribed. And the transcripts were reviewed in relation to the questions asked and associative themes (thematic content analyses).^26,29^ The same approach was used for the open-ended questionnaire items. This study deployed an inductive, bottom-up approach, as well as a more deductive, top-down approach, to analysing the data, and deployed categories derived from existing literature.

Completed questionnaires were coded using SPSS with the data being subjected to descriptive statistical treatment and not inferential statistics because the sampled clinics cannot be used to infer properties of all health facilities in the country. Additionally, demographic data and questionnaires were given descriptive statistical analysis.

The initial coding, descriptive statistics derived from questionnaire and analysis of data was done by the first author while co-authors verified the codes and themes to ensure that they were reflective of the data. This was an iterative process, which included the analysis of free responses sections across the questionnaires. The preliminary findings of this study were presented and scrutinized at a study workshop convened virtually by the IAS – the International AIDS Society – in June 2021. This workshop was attended by research experts from Kenya, Malawi and Zambia, as well as from the United States, who gave feedback on this study. This feedback assisted in the further analysis of data, thereby ensuring rigour in this research.

### Ethical Approval and Informed Consent

The University of Zambia Humanities and Social Sciences Research Ethics Committee in Lusaka approved the study (approval ref no: HSSREC 2021 – FEB – 007). Permission to conduct the research on HIV stigma was also obtained prior to going into the health facilities from the IAS in Geneva, Switzerland, and the Zambian Ministry of Health through the National Health Research Authority in Zambia (permit ref no: NHRA00002/26/03/2021). This study was conducted in accordance with some guidelines and procedures set out in the Declaration of Helsinki. All participants gave written informed consent before participating in the study.

## Results

The first part of the results section presents demographic characteristics of participants. The second part shows the impact of ART on stigma; the third looks at continued experiences of stigma for healthcare workers living with HIV; the fourth explores quiet navigation of stigma; and the fifth concerns stigma reduction strategies, including codes of conduct. Participant demographic features are presented in Table 1.

**Table 1.** Participant Characteristics (N = 60).

*Feature*	*Description*	*Frequency*	*Percent*
*Gender*	Men	14	23
*(n* *=* *60)*	Women	42	70
* *	Trans/other	4	8
* *			
*Age*	18–24	3	5
*(Responses n* *=* *60)*	25–34	24	40
* *	35–44	19	32
* *	45–54	12	20
* *	55 +	2	3
* *			
*Sexualities*	Bisexual	3	6
*(Responses n* *=* *53)*	Heterosexual	43	81
* *	Prefer not to say	7	13
* *			
*Education level*	Secondary	6	11
*(Responses n* *=* *57)*	Tertiary	51	89
* *			
*Role*	Senior medical	4	6
*(Responses n* *=* *60)*	Mid-level medical nurse	41	68
* *	Admin staff	3	5
* *	Support staff	7	12
* *	Service users	4	7
* *	Other	1	2
* *			
*Time in healthcare*	Less than 1 year	7	12
*(Responses n* *=* *60)*	2 − 5	19	32
* *	6 − 10	17	28
* *	11 − 15	13	22
* *	16 +	4	6
* *			
*Residence*	Urban	23	38
*(Responses n* *=* *60)*	Peri-urban	37	62

### Effects of ART on Reducing Stigma

Forty-eighty people (96%) in the questionnaire agreed that treatment was changing the level of HIV stigma. Interview participants indicated that ART has had a big impact on stigma reduction. A common response in qualitative parts of the questionnaire was that ART ‘brings hope’, which participants linked to HIV no longer being a death sentence. They described advanced HIV disease as being less visible and therefore generating less stigma. A popular response in the questionnaires was that people on ART can live a ‘normal life/healthier life’. Asked about their first thoughts linked to HIV, ‘ART/treatment’ was also the most mentioned term. Responses were given, for example, around colleagues or some health workers who were accessing their treatment openly.

Even recently, we were having a working lunch discussion with health providers and one of them said, ‘Let me go and take my ARVs before I eat this nice food.’ She was just open and there was no rolling of eyes like there might have been before. (Key informant interview)

Other key informant interviewees also confirmed this development:

There was one time when a clinic was very busy and one of the staff spoke to the pharmacist to say, ‘I will have to pick up my ARVs later. I am so busy.’ Then the pharmacist just waited for her and the clinician – everyone knew and there was no hiding it. (Key informant interview)

When asked if ARVs reduced stigma towards people living with HIV, the large majority (96%) agreed or strongly agreed. This was in part attributed to the improved physical health of being on ART that does not make a person living with HIV appear frail.

The role of treatment in stigma reduction was related to reduced fear of HIV acquisition and reduced HIV-related shame by questionnaire participants. A total of 28% agreed that hospitals or other medical environments were not physically safe in terms of HIV transmission, for example, through acquiring the virus due to unsafe surgical procedures. No participants referred to U = U in both interviews and questionnaire free-response sections.

### Persisting Stigma

A total of 43% of questionnaire respondents gave fear of labelling and being judged by others and themselves as the main reason for not testing and seeking treatment for HIV. Despite participants saying stigma has reduced, labelling and judgemental attitudes were said to have remained unchanged. For example, the majority of participants noted that earlier in the epidemic, HIV was associated with ‘promiscuity’ and multiple sexual partners. Although the availability of ART has reduced stigma because people are living healthy lives and taking their drugs freely, these associations with transgressive sexuality were continuing to generate stigma. One example included a newly transferred member of health staff ‘suspected’ of being HIV positive who had arrived with a ‘reputation’ of being ‘promiscuous’. Key informants in the interviews said that such attitudes resulted in many health workers not wanting others to know about their status.

For most interview participants with unknown HIV statuses, silence and fear of disclosure emerged. In explaining the lack of openness about their HIV status at work, a common fear was that trust and confidentiality may be broken. Where stigma was being quietly managed, several informants in interviews reported that they had observed some healthcare workers who just told the sister-in-charge or the counsellor about their HIV status if they were comfortable with them as supervisors. This then enabled them to access services discreetly.

The majority of participants in both the interview and questionnaires indicated issues around broken confidentiality, such as workers checking colleagues’ files, and hearing comments such as, ‘It [her behaviour] is because she is sick.’ Responses also included reports of blame and of pushing colleagues away, saying, ‘Go and work there ….’ Most participants stated that experiences of stigma made health workers less willing to test for HIV and seek treatment. Fear and anxiety about what people will say or think about being seen at the ART clinic have a major impact.

Lack of confidentiality was a crucial element in questionnaire responses. In all, 72% of questionnaire respondents endorsed the problem of lack of privacy among healthcare workers. A total of 60% of participants agreed with the statement that if they were living with HIV, they would worry about some health workers not keeping information confidential.

Thirty-six participants indicated worrying about breaches of privacy by some healthcare workers. One suggested that some healthcare workers feared disclosing their status due to anticipated stigma, which can also lead to seeking services in distant clinics for privacy, rather than within a workplace or nearby facility.

### ‘Quiet Navigation’ of Stigma

Many participants spoke in the questionnaire's free-response section about how stigma was being managed by what we are calling ‘quiet navigation’ in health facilities. Key informants in the interviews stated that by quiet navigation, we avoid confronting stigma for others related to health service access by finding strategies that are often informal and allow privacy to be upheld. For example, by creating flexible services to meet the needs of staff, living with HIV assures confidentiality. Special arrangements often made for healthcare workers, such as taking ARVs to homes of staff, was mentioned in interviews. Private testing was also offered to those on or needing treatment:

The first health worker came to me and said, ‘I just want to check myself.’ But I asked her, ‘Can we talk first before you test?’ So, I did the pre-test counselling and she tested but then she said she did not want the results straight away. I accepted this. She came back two days later – I did the post-test counselling to help her prepare. When we saw the results were positive, she went very quiet and then tears started falling … I suggested she choose one health worker, one nurse who she was happy to work with. I also said if she preferred to go to a different health centre, she could do that. But I encouraged her to stay here so that we could help her. She didn't want anyone to know – we discussed who she could trust and I thought it would be good if she could at least tell one colleague. And then we agreed how she could come to pick up her medicine when there were no patients around, and check her vitals*.* (Key informant interview)

In the extract above, we see that quiet navigation of stigma includes provision of private appointments outside normal hours. Other elements include delivery of ARVs in plain packages and conducting blood tests at home. We must note here that all these provisions were being made for a colleague**.** That colleague has a level of social and perhaps (as a medical professional) cultural capital not available to other service users. We can also notice from the comment that a change of location for HIV services is one way of avoiding stigma.

Talking about the issue of ART access, one interviewee said that fast tracking of services was considered for health workers ‘if you know they are a health worker’, supporting the preferential access to stigma-aware services described above. The interview results showed seven mentions of examples of how health workers living with HIV accessed counselling support and collection of ARVs outside of normal clinic hours. Home delivery of ART drugs was noted in interviews as an example of quiet navigation related to service users generally.

Participants indicated a variety of specific provisions that could in future reduce HIV stigma in healthcare settings. In some cases, this possibility arose from treatment success. Participants generally expressed positive views about HIV treatment. For example, one of the key informants stated that people living with HIV were healthier because of treatment: ‘Now we have a person who is HIV positive and on treatment looking and being healthier than those who are HIV negative.’ The effectiveness of HIV treatment was seen as an opportunity to work towards stigma-free services, as well as having already reduced stigma.

However, some participants suggested that the challenges of HIV stigma can also be addressed by policies and guidelines of which staff are not aware and which are hardly implemented. In one case, a senior member of the nursing staff assumed no stigma policies because they had not heard of them, and assumed also that posters they saw ‘around’ were not connected to any institutional policy: ‘We do not have a policy here, but I have seen a few posters stuck around talking about stigma.’ (Key Informant Interview)

Moreover, the stigma associated with treatment in health facilities was linked to negative images of health facilities being unfriendly environments where staff living with HIV – let alone service users – cannot be open without risk. One participant suggested that privacy and confidentiality should be assured in healthcare settings for healthcare workers with HIV and non-healthcare worker service users alike.

The need to disclose HIV status was often reported in the questionnaire as associated with anticipated stigma. Not all participants mentioned or supported unrestricted disclosure and general status openness. The majority (n = 45) of questionnaire respondents thought less-assured privacy and confidentiality would suffice to provide a ‘good-enough’ support network of people disclosed to, as indicated in the response of the counsellor in the previous section.

### National Standards, Guidelines and Policies on HIV Stigma

Key informant interviews in both senior and lower-level roles in health facilities emphasized the need for anti-stigma guidance to be implemented through policies in their workplaces. At the same time, they reported low awareness of any existing policies around HIV stigma. A ‘code of conduct’ was mentioned in interviews as a reminder and an awareness that ‘it is not OK to stigmatize’, but there seemed to be no consistent source of a code of conduct as participants in both interviews and questionnaires could not indicate whether it was from the Ministry of Health or health facility management.

The only guidelines identified through interviews seemed to be related to an outdated 2004 HIV Workplace Policy that has apparently not been reviewed since dispersal. These national standards were said to contain treatment guidelines; for example, many mentioned ‘Access for All’, and stigma was included as a ‘cross-cutting issue’.

Most of the key informants specifically mentioned the lack of HIV stigma guidelines for healthcare workers rather than for workers generally or the general population. Further, the key informants argued that there were no clear policies to enable healthcare workers to test for HIV and access ARVs in the context of a workplace free from stigma. Others said that the fast-tracking initiatives were not formalized and were practiced in only a few health facilities. However, key informants also relayed that stigma was covered and discussed in management meetings and when staff made complaints, these were taken up by relevant office bearers within a health facility.

Health facility managers and HIV focal point people were reported to be responsible for policy development and dissemination in clinics. However, most key informants said that it was usually counsellors and gender-based violence representatives who were left to be responsible without support and guidance on anti-stigma work policy enforcement from senior health staff or higher management. From both the interviews and questionnaires, there were neither clear, established reporting mechanisms for stigmatizing experiences, nor ways of seeking official redress mentioned within health facilities structures.

### Reducing or Ending Stigma in Healthcare Settings

Respondents identified several elements that they thought would help end stigma in healthcare settings, including training, integration of services and delegation of responsibility to challenge stigma to all staff.

No participants had received training that focused purely on stigma reduction. In spite of several workshops that participants had attended, no specific stigma reduction training was taking place. Most key informants reported that the topic of stigma had been integrated into many trainings but thought that specific training would be more useful:

I think if we had stigma training across the board, it would be great; to know that all health workers received training to recognize stigma, rather than just tag it onto other trainings. (Key informant interview)

Most key informants highlighted status loss or professional embarrassment as a stigma form for health workers living with HIV. Integration was mentioned as being effective in reducing stigma that comes with designated HIV service delivery:

The best solution would be a fully integrated service: you start at OPD (the outpatient department) just like any other patient, rather than having separate services – you should be embraced by the service, not feel pushed to one side. A lot of the problem is that those who plan the programme never consult those who will be using the services – and service users have a lot to say, a lot of good ideas and suggestions. (Key informant interview)

Key informants conveyed a sense that tackling stigma is the responsibility of everyone. In explaining the aim of ending stigma, collective effort by healthcare personnel was at the centre of participants’ desire for the future: ‘Us, the health workers, need to make this (stigma-free services) happen’. (Key informant interview)

## Discussion

Data from this study suggest that ART has positively changed stigma experiences in healthcare settings. The responses from participants show that HIV treatment is an effective tool in reducing stigma. Our findings and other Zambian research (see Chitambala et al) indicate that improved physical health and disease invisibility was a premise upon which stigmatizing behaviours were minimized among healthcare staff and service users.^18^

The other major themes in the findings are that although effective HIV treatment reduces stigma, there is persistent stigma reported by healthcare workers and that the socially unsafe health facility environment leads to the theme of ‘quiet navigation of stigma’ for people living with HIV who are healthcare workers themselves*.* Other key findings include HIV-related stigma being shaped by HIV treatment success. The absence or presence of up-to-date and specific national standards or guidelines has a bearing on ending or reducing stigma efforts. The training received or not acquired by healthcare workers is influencing how stigma is dealt with in clinics, as well as determining the effectiveness of existing interventions against stigma in healthcare settings.

It is clear from the findings that some historical experiences of HIV still fuel fear about transmission, and this echoes other research from African contexts.^26^ Further, ART and the technologies around it undergird positive attitudes by providing a health setting that is conducive to knowledge acquisition, stigma reduction and action. However, participants in this study reflected that stigma in healthcare settings is still a major concern because, as a social process, it can powerfully impede people living with HIV from accessing services and can result in lack of adherence to ART due to labelling and discrimination sometimes occurring in health facilities.^31^ Similar findings were reported in other regional studies. Stigmatizing behaviour in health facility settings happens at both the structural and individual levels, and this is experienced by healthcare workers living with HIV, as well as service users.^6^ Some healthcare workers were said by participants to opt to access HIV services in a different health facility due to fear of being stigmatized and lack of confidentiality and privacy in their own workplaces.

The way HIV services are situated in healthcare settings demonstrates inequalities of spatial organization, like having designated places, windows or rooms for ART collection, which results in a lack of confidentiality.^6^ Structural issues, such as class, play a part in the failure to prioritize healthcare workers living with HIV, which leads to internalized and anticipated stigma, as also noted by Stangl and colleagues.^9^ We show that willingness to seek HIV services in health facilities is dependent on confidentiality, trust and disclosure restrictions. The study revealed that HIV stigma experiences at health facilities were frequently due to lack of privacy and trust among and between healthcare workers and service users.

Nonetheless, healthcare workers have invented ways of avoiding stigma through ‘quiet navigation’. For example, the enabling role of colleagues made such actions as dispensing out of hours possible; it also enabled the healthcare workers living with HIV themselves to quietly navigate stigma. They have also advanced the need for targeted training, integrated services and individuals taking responsibility to reduce stigma.^30^ Flexible services and other forms of ‘quiet navigation’, such as home delivery of ARVs, are important aspects of what happens in healthcare facilities. These could be the foundation for other kinds of anti-stigma work and, indeed, they are in themselves important in showing that stigma is wrong and can be combatted even in difficult circumstances. They also seem like strategies that are very sensitive to the particularities of individual lives – a sensitivity that is itself important. This finding on quiet navigation of stigma is a major contribution of this study as it had not been explored by any of the works that were surveyed.

Moreover, healthcare workers are generally unaware of the existence of stigma reduction policies or guidelines, and the available guideline we found was very outdated. Tackling stigma in healthcare is complex, especially when there are no clear policies; this finding is supported by Nyblade and colleagues.^26^ We found that there was a recognition of lack of and non-enforcement of up-to-date standards, guidelines and codes of conduct that should be employed against stigmatizing practices. From the findings, we can see that ending stigma in health facility settings and promoting the agency of all healthcare workers as individuals depends on strong guidelines and capacity-building programmes, such as training for health personnel. Besides lack of knowledge of guidelines and their enforcement, we found that there was also a lack of training for healthcare workers on stigma prevention and reduction.

There is no investment in stigma reduction programmes for healthcare workers. Thus, there is an urgent need for the Zambia health system to devise, avail, implement and enforce stigma reduction policies and guidelines. Training healthcare workers on stigma reduction is also crucial. The current inadequate stigma reduction training offers an opportunity for rolling out of several globally recognized toolkits that exist, but may need re-standardization and/or reworking for specific national and local contexts, as also reported by the Global Network for People living with HIV,^27^ Biemba and others.^28^

Our findings suggest several routes through which healthcare facilities can reduce HIV stigma. A total of 80% of participants from both interviews and questionnaires in this research reported how it was possible to reduce stigma in healthcare settings. In spite of available materials on stigma, there was a strong aspiration for more information that is context based, coupled with a sense of responsibility that will instil ethical behaviour in health facilities. This finding is similar to that of Mulubale, Rohleder and Squire,^2^ who did a study on HIV stigma messaging using comedy.

Also, to increase the resistance for anticipated, internalized and external stigma, peer communication, community-based healthcare (such as delivery of ARVs to homes, policy direction, community care and framing HIV anti-stigma messages as mental health promotion rather than deviant intervention routes) may be essential in dealing with discrimination of being seen at ART departments in clinics, as mentioned by interview participants and found in studies by Mulubale,^8^ Bond and others.^6^

Finally, interactions at health facilities provide a solid foundation for HIV treatment adherence, which, given the prevalence of HIV in Zambia, is important to address. Frequent disclosure by some healthcare workers could be destigmatizing for those living with HIV, encouraging testing and treatment, and could help reduce problems of HIV for groups vulnerable to HIV, as shown by Mackworth-Young, Bond and Wringe,^16^ as well as Biemba and colleagues.^28^

## Research Limitations

This research is not devoid of limitations. Undoubtedly, COVID-19 restrictions in Zambia made the activities of this work challenging. However, the periodic easing of the lockdown allowed the fieldwork to proceed with data saturation being achieved following masking, physical distancing and hygiene regulations.

Permission from the National Health Research Authority (NHRA), as well as travel demands for research, created some time overspills. Conducting research in clinics requires several authorizations alongside ethical approvals. Delays in being granted permission from NHRA contributed to modification of the research timeframe, which was extended and caused some logistical constraints. Nonetheless, this procedural issue did not affect the findings of the study in any way.

The scientific limitations of this study can be hinged on the sample biases. Of the several clinics in the five provinces that were covered, only one health facility was sampled per region, which could mean that the findings presented here are less generalizable. The imbalance between 10 key informant interviews on which this study relied a lot and 50 questionnaires speaks to the qualitative bias of the findings represented here. The free association was only possible through the free responses section of the questionnaire. Having four service users (non-healthcare workers) as participants and a small number of men and using descriptive statistics makes it hard to generalize about some of the findings. Similarly, the sample size and selection of participants (healthcare workers from different units) within clinics may have some responder bias due to workplace restrictions and perhaps differences in accuracy to retrieve past events and experiences of stigma based on positions held and roles played at the health facilities. To deal with recall limitations, sufficient time was allowed and questions were repeated during interviews.

Finally, stigma issues in health facilities are both complex and organizational. The way clinics handle or deal with HIV-related stigma manifestations differs based on context and patterns of inequalities within a given location. This mixed methods study did not address such issues fully; nor was it designed to explore limitations of existing projects in the area of HIV stigma generally.

## Conclusions

This study has indicated that access to treatment has changed perceptions of HIV and reduced stigma manifestations, such as negative attitudes, stereotyping, discrimination and gossip. This paper has described ‘quiet navigation’ as a measure being used by health workers living with HIV to deal with some of the persistent stigma, which still exists through associations of HIV with promiscuity and offensive labelling. The article highlights local initiatives, such as delivering ART to clients’ homes, which help combat or avoid stigma by managing it through flexible services and ‘quiet navigation’.

The study revealed that lack of confidentiality, and fear of lack of confidentiality, play a critical role in fuelling fear of disclosure and hampering access to testing and treatment. Privacy was also found to be valuable in relation to fear of confidentiality breach in the process of seeking services in health facilities. The issue of whether HIV services can be mainstreamed came out as an intervention. Participants reported flexibility and special arrangements for healthcare workers in relation to stigma of ‘being seen’ (at ART clinics).

This research's empirical and theoretical implications of findings are that increased treatment access has helped normalize living with HIV in health facility settings. Biomedical improvements have contributed to hopeful messages and stigma navigation through representations of lives with HIV as normal healthy lives. However, more needs to be done to address stigma as it is still a major concern in healthcare facilities and for healthcare workers living with HIV within Zambia.
